# Correction: Diffusion MRI reveals in vivo and non-invasively changes in astrocyte function induced by an aquaporin-4 inhibitor

**DOI:** 10.1371/journal.pone.0235581

**Published:** 2020-06-25

**Authors:** The PLOS ONE Staff

The first author's name is spelled incorrectly. The correct name is: Clement Debacker. The correct citation is: Debacker C, Djemai B, Ciobanu L, Tsurugizawa T, Le Bihan D (2020) Diffusion MRI reveals in vivo and non-invasively changes in astrocyte function induced by an aquaporin-4 inhibitor. PLoS ONE 15(5): e0229702. https://doi.org/10.1371/journal.pone.0229702

There is an error in affiliation 1 for all authors. The correct affiliation 1 is: NeuroSpin/Joliot, CEA-Saclay Center, Gif-sur-Yvette, France. The publisher apologizes for the errors.
