# Supplementary figures and images for: Diffusion MRI reveals in vivo and non-invasively changes in astrocyte function induced by an aquaporin-4 inhibitor
Source: PLoS One. 2020 May 15;15(5):e0229702. doi: 10.1371/journal.pone.0229702 (PMC7228049; doi:10.1371/journal.pone.0229702)

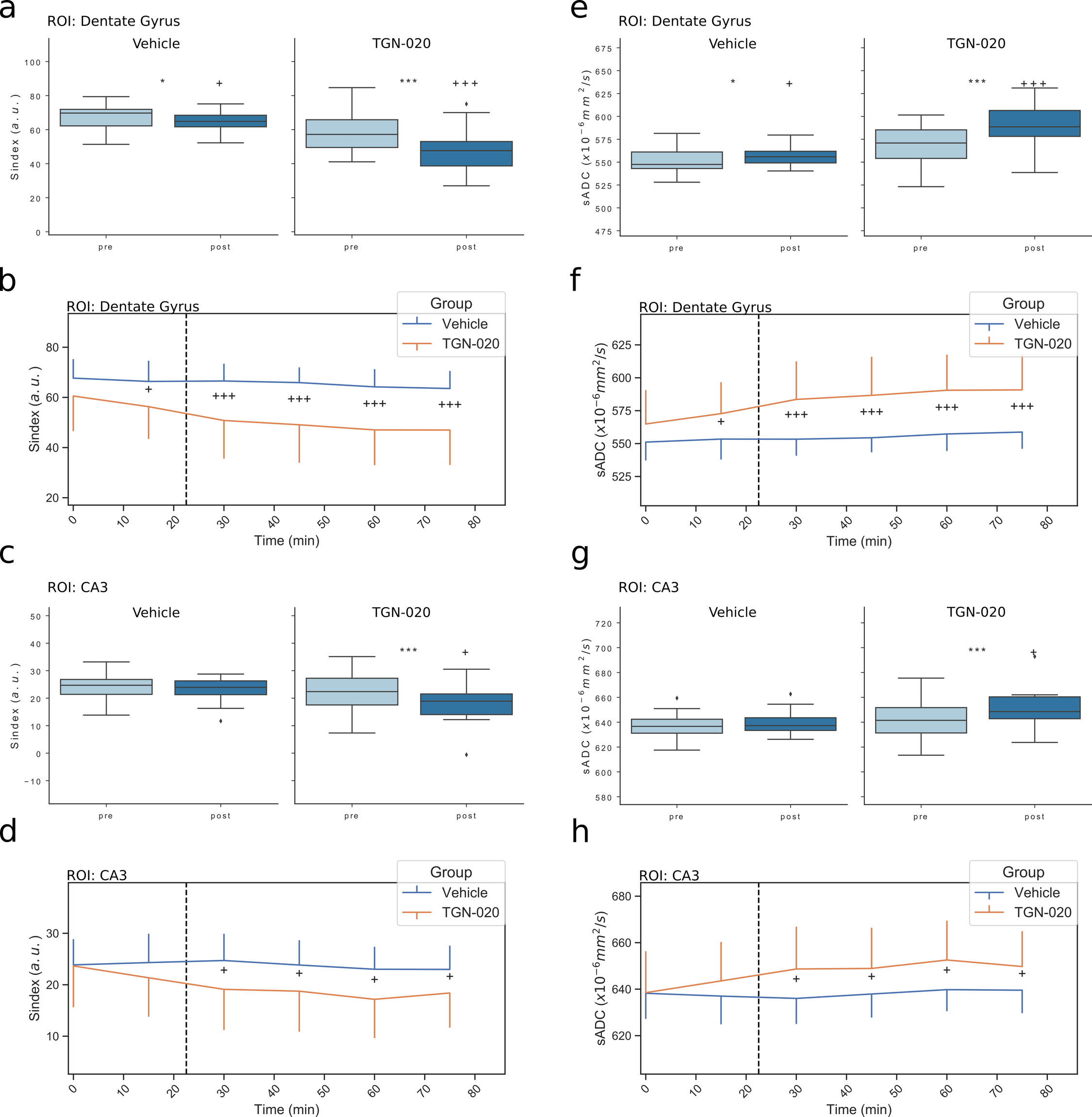

Supplement: S1 Fig — Boxplot and time course of Sindex in the DG (a: boxplot, b: time course) and in the CA3 (c: boxplot, d: time course). Boxplot and time course of sADC in the DG (e: boxplot, f: time course) and in the CA3 (g: boxplot, h: time course). Left, Vehicle group; right TGN-020 group in each figure. Pre, pre-injection (light blue); post, post-injection (dark blue) of vehicle or TGN-020 group in boxplot. *: p<0.05, **: p<0.01, ***: p<0.001 are the result for the paired t-test between pre and post-injection for each group. +: p<0.05, ++: p<0.01, +++: p<0.001 are the result for a t-test with posthoc correction for multiple comparison between post-injection of the two groups. Diamonds represent outliers, a point is defined as an outlier if its value is below Q1–1.5×IQR or above Q3 + 1.5×IQR, where Q1 is the first quartile, Q3 the third quartile and IQR the interquartile range. Blue line is vehicle group and orange line is TGN-020 group. Error bar shows standard deviation. The dashed line represents the injection time. +: p<0.05, ++: p<0.01, +++: p<0.001 by two sample t-test between the two groups for each time point. Note: The voxel count in DG was very small (85 versus 181 in CA3) resulting in very noisy data. The apparent significant difference in sADC and Sindex values between the TGN-020 and saline groups one point before baseline (b, f) might result from an underestimation of the standard-deviation. The difference becomes largely more significant after injections, overcoming any uncertainty in standard-deviation estimates. (TIF) [file pone.0229702.s001.tif]
