# Supplementary material for: Diffusion MRI reveals in vivo and non-invasively changes in astrocyte function induced by an aquaporin-4 inhibitor
Source: PLoS One. 2020 May 15;15(5):e0229702. doi: 10.1371/journal.pone.0229702 (PMC7228049; doi:10.1371/journal.pone.0229702)
Supplement: Attachment — Submitted filename: 200303-responses_to_reviewers-REV.docx [file pone.0229702.s003.docx]

*Reviewer #1: Authors responded to the raised issues and addressed reviewer’s concern.

One point that remained unaddressed is the general assumption. The author states that “The aim of our work is to show that the modulation of astrocyte activity by TGN-020 which is known as a AQP4 channel blocker can be monitored with diffusion MRI and in particular the Sindex, that’s all.”

But throughout the paper the text suggests that that’s not all. For example, the abstract says

“The Glymphatic System (GS) has been proposed as a mechanism to clear brain tissue from waste. Its dysfunction might lead to several brain pathologies, including the Alzheimer’disease. A key component of the GS and brain tissue water circulation is the astrocyte which is regulated by acquaporin-4 (AQP4), a membrane-bound water channel on the astrocytic end-feet.”

Well, clearly the author is relating this to glymphatic system. Yet, relating this to glymphatic system is not the main concern. If one can measure astrocyte activity, it’s not unreasonable to relate it to glymphatic system. The main concern is that AQP-4 is not only involved in modulation of astrocyte activity. It is also involved in the brain inflammatory response and also in the regulation of the extracellular volume. Both of these could affect diffusion signal. Blocking AQP-4 could lead to inflammation or changes in the extracellular fluid which affect the diffusion signal. Therefore, what you observe here may have nothing to do with the astrocyte activity. This limitation should be addressed.

PS. Regarding DG, if the data is noisy, it should lead to higher standard deviation not a systematic group mean difference*

- The statement about the Glymphatic System in the abstract and the introduction is only to explain our general motivation to conduct our study of the potential of diffusion MRI to monitor astrocyte activity. It is not a claim that the GS exists (we believe we have been careful in the manuscript about this fact). We only briefly review the literature to shade light on the context of the study and indicate why investigating astrocyte activity might be important. We are aware that TGN-020 effects are not specific to astrocytes, as stated in the manuscript. Effects on the brain inflammatory response can be discarded given the time course of the effects we have observed. Regarding the extracellular space (ISF) effects on the diffusion MRI would result only from changes in diffusion hindrance/tortuosity (fluid flow is well too slow to result in IVIM effects, this has been shown by other groups, furthermore the Sindex was purposely calculated using the lowest key b value 250s/mm² to make residual IVIM effects completely negligible). Hence, variations in extracellular space shape and volume only reflect changes in the local cellular background (a kind of negative imprint). The decrease in Sindex (and sADC increase) points out to decrease in the amount of diffusion hindrance from astrocytes (which is by far the dominant cell type in the regions where we observed the largest response). As stated in the discussion “Based on established diffusion MRI mechanisms [28] this hindrance decrease suggests an astrocyte volume reduction [43,44] associated with an increase of the ISF (were diffusion is tortuous) [45] or an increase in astrocyte membrane permeability and water exchange”. We believe that this statement about the mechanism leading to our observation is correct and broad enough, and the manuscript was not changed.
- Regarding the DG, the data are, indeed, very noisy given the very small voxel count. The apparent group difference may just be coincidental as the standard-deviation cannot be accurately estimated precisely given the small voxel count. We realize that this might be puzzling, but, for scientific integrity, we decided not to “hide” those results but to show them. A note has been added to the caption of SuppL Fig.1.
